# Association Between Coronary Artery Disease and Plasma Omentin-1 Levels

**DOI:** 10.7759/cureus.17347

**Published:** 2021-08-21

**Authors:** Priya Bai, FNU Abdullah, Maham Lodi, Mayur Sarhadi, Anum Dilip, Sibghatullah Shahab, FNU Yasir, Maha Jahangir

**Affiliations:** 1 Internal Medicine, People's University of Medical and Health Sciences for Women, Nawabshah, PAK; 2 Neurology, Jinnah Sindh Medical University, Karachi, PAK; 3 Internal Medicine, Jinnah Sindh Medical University, Karachi, PAK; 4 Internal Medicine, Jinnah Medical and Dental College, Karachi, PAK; 5 Internal Medicine, Baqai Medical University, Karachi, PAK; 6 Internal Medicine, Dow University of Health Sciences, Karachi, PAK

**Keywords:** cardiovascular disease, coronary artery disease, cad, omentin-1, intelectin-1

## Abstract

Introduction: Omentin-1 is secreted from visceral adipose tissue that contributes to chronic inflammatory diseases’ pathogenesis, including cardiovascular events. In this case-control study, we will determine the association between plasma omentin and coronary artery disease (CAD).

Methods: This is a case-control study, conducted from June 2020 to April 2021 in the cardiology unit of a tertiary care hospital in Pakistan. Patients diagnosed with CAD (n = 300) within the last six months were included in the study. Another 300 participants without CAD and with similar demographic profiles were included in the control group. A blood sample of 5 ml was drawn from participants of both groups and sent to the laboratory to test for plasma omentin level.

Results: Plasma omentin level was significantly lower in patients with CAD compared to the patients without CAD (61.21 ± 10.21 ng/dL vs. 95.22 ± 12.21 ug/L; p-value: <0.0001). In both genders, the plasma omentin-1 was lower in patients with CAD compared to patients without CAD (p-value: <0.0001).

Conclusion: The present study revealed a negative association between omentin-1 and CAD. We speculate that low levels of omentin-1 might play a role in the pathogenesis of atherosclerosis. Therefore, plasma omentin-1 can be a potential biomarker to predict the development and progression of CAD.

## Introduction

Coronary artery disease (CAD) is a cardiovascular disease (CVD) that is found to be the leading cause of death in both developed and developing worlds [1]. It is characterized by atherosclerotic changes, which are inflammatory in nature. Along with genetic and environmental factors, lifestyle has been found to play an important role in the investigation of the clinical phenotype of CVD. Several risk factors have been associated with the development of CAD, such as hypertension, smoking, diabetes, and hyperlipidemia [2-5].

Measurement of biomarkers has revolutionized the work-up of patients with suspected cardiovascular disease. The most widely used are the natriuretic peptides in the diagnosis and prognosis of heart failure and cardiac troponins in the diagnosis of acute myocardial infarction.

A broad range of biomarkers is associated with cardiovascular risks. The natriuretic peptides and cardiac troponins are among the most widely used contemporary cardiovascular biomarkers that help in the diagnosis and prognosis of CVDs. The scientific advances have led to the discovery of a vast array of novel biomarkers associated with cardiovascular risks, including myeloperoxidase, lipoprotein-associated phospholipase A2, fibrinogen, trimethylamine N-oxide, and cystatin C [6]. Another novel biomarker for CAD is plasma omentin-1 [7].

Like other adipokines, omentin-1 is expressed from visceral adipose tissue that contributes to chronic inflammatory diseases’ pathogenesis, including cardiovascular events. Several studies have demonstrated that circulating omentin-1 levels are associated with several metabolic risk factors, such as high blood pressure, dyslipidemia, and glucose intolerance [7]. However, there are contradictory data available. In his study, Saely et al. state that patients within the high omentin group reported significantly more cardiovascular events than patients in the low omentin group [7]. Askin et al. stated that a decrease in the omentin levels is an important predictor of CAD and its severity [8]. Zhou et al. also found a correlation between higher plasma omentin-1 level and better coronary collateral circulation development [9].

Currently, very little data are available globally and regionally related to the correlation between plasma omentin and CAD. In this case-control study, we will determine the association between the two.

## Materials and methods

This is a case-control study, conducted from June 2020 to April 2021 in the cardiology unit of a tertiary care hospital in Pakistan. CAD was defined as damage to the major blood vessels of the heart secondary to atherosclerotic changes and inflammation. Patients diagnosed with CAD (n = 300) within the last six months were included in the study. Diagnosis of CAD was made by an interventional cardiologist via angiography. Another 300 participants without CAD and with similar demographic profiles were included in the control group. Informed consent was taken from each participant after the entire process was explained to them. Case and control groups both were enrolled using a consecutive convenient non-probability sampling technique. The process of enrollment was started only after the approval from the ethical review board of the institute.

After the process of enrollment, participants were asked about their age, gender, body mass index (BMI), and relevant history. A blood sample of 5 ml was drawn from participants of both case and control groups from the cubital vein and sent to the laboratory to test for plasma omentin level. The blood sample was collected in the morning to avoid diurnal fluctuation. Plasma omentin was assessed using an enzyme-linked immune-sorbent assay.

Data were analyzed using the statistical software Statistical Packages for Social Sciences (SPSS) v. 23.0 (IBM Corporation, Armonk, New York). Chi-square and unpaired t-test were applied to compare the parameters of two groups. A p-value of less than 0.05 meant that there is a significant difference in the value between the two groups and the null hypothesis is void.

## Results

In this study, no significant difference in age and gender distribution was found between the two groups. Risk factors, including diabetes, hypertension, smoking, and BMI more than 25 kg/m^2^, were comparable in both groups and no significant difference was found between them (Table 1).

**Table 1:** Characteristics of the study participants BMI: body mass index; CAD: coronary artery disease; kg/m2: kilogram per square meter; NS: nonsignificant; SD: standard deviation.

Characteristics	Patients with CAD (n = 300)	Patients without CAD (n = 300)	p-value
Age in years (mean ± SD)	52 ± 10	53 ± 10	NS
Male	181 (60.33%)	186 (62.0%)	NS
Hypertension	281 (93.66%)	283 (94.3%)	NS
Diabetes	154 (51.3%)	160 (53.3%)	NS
Smoking	112 (37.3%)	116 (38.6%)	NS
BMI more than 25 kg/m^2^	131 (43.6%)	128 (42.6%)	NS

Plasma omentin level was significantly lower in patients with CAD compared to the patients without CAD (61.21 ± 10.21 ng/dL vs. 95.22 ± 12.21 ng/dL; p-value: <0.0001). For males, plasma omentin-1 was lower in patients with CAD compared to patients without CAD (61.78 ± 10.12 ng/dL vs. 95.23 ± 12.17 ng/dL; p-value: <0.0001). Similarly, plasma omentin-1 was lower in female patients with CAD compared to their counterparts (60.98 ± 10.02 ng/dL vs. 94.80 ± 12.04 ng/dL; p-value: <0.0001) (Table 2).

**Table 2:** Gender-wise comparison of omentin-1 levels in patients with and without CAD CAD: coronary artery disease; ng/dL: nanogram per decilitre; SD: standard deviation.

Omentin-1 levels (mean ± SD ng/dL)	Patients with CAD (n = 300)	Patients without CAD (n = 300)	p-value
Total omentin-1	61.21 ± 10.21	95.22 ± 12.21	<0.0001
Male	61.78 ± 10.12	95.23 ± 12.17	<0.0001
Female	60.98 ± 10.02	94.80 ± 12.04	<0.0001

## Discussion

Our study demonstrated that patients with CAD reported lower levels of omentin-1 than those who did not have CAD. Literature has enough evidence in concordance with our results. A cross-sectional study conducted by Shang et al. included metabolic syndrome (MetS) patients to analyze the association between omentin-1 level and CAD [10]. It was suggested that MetS patients with CAD were reported to have lower levels of omentin-1 compared to those who did not have CAD. Additionally, omentin-1 showed a negative association with the presence and angiographic severity of CAD [10]. In recent years, studies support the evidence that omentin-1 could potentially be the reason behind the pathology of atherosclerosis. Liu et al. also established a negative link between omentin-1 level and carotid atherosclerosis in MetS patients [11].

Adipose tissues primarily help in conserving energy and also demonstrate endocrine functions in producing and secreting several bioactive adipokines that further lead to CVDs [12]. Omentin-1, also known as intelectin-1, is abundantly expressed in intra-abdominal fat [13]. There is evidence that obese patients demonstrate low levels of plasma omentin-1, and its levels are negatively related to BMI, waist circumference, leptin levels, and insulin resistance [14]. Furthermore, omentin-1 is not positively linked with the presence and angiographic severity of CAD in MetS patients [10]. It also imposes neutralizing effects on atherosclerosis and ischemia-induced revascularization [15-17].

In light of the above findings, future studies including a larger sample size should be carried out to confirm our results. Our study suggests that plasma omentin-1 levels should be assessed at regular intervals in at-risk patients or those who have underlying cardiac issues. This would help in better management and monitoring of heart-related problems.

To the best of our knowledge, this was the first study that explored the association between plasma omentin-1 and CAD in a local setting. However, since the study was conducted in a single institute, sample size diversity was limited. Similarly, since it was a case-control study, the causal relationship between the two could not be confirmed. Therefore, more studies, including large and diverse sample sizes, should be conducted to explore and establish the association between plasma omentin-1 and CAD.

## Conclusions

In conclusion, the present study revealed a negative association between omentin-1 and CAD. We speculate that low levels of omentin-1 might play a role in the pathogenesis of atherosclerosis and adequate levels of omentin-1 are beneficial in maintaining a healthy state of the heart and its function. Therefore, plasma omentin-1 can be a potential biomarker to predict the development and progression of CAD.
